# Numerical Study on the Development of Adiabatic Shear Bands During High Strain-Rate Compression of AISI 1045 Steel: A Comparative Analysis Between Plane-Strain and Axisymmetric Problems

**DOI:** 10.3390/ma17215286

**Published:** 2024-10-30

**Authors:** Konstantina D. Karantza, Dimitrios E. Manolakos

**Affiliations:** Laboratory of Manufacturing Technology, School of Mechanical Engineering, National Technical University of Athens, Heroon Polytechniou 9, 15780 Athens, Greece; konstantinakarantza@mail.ntua.gr

**Keywords:** adiabatic shear bands, shear localization, plane strain compression, axisymmetric compression, thermal softening, damage softening, coupled analysis, LS-DYNA

## Abstract

This work studies numerically the development of adiabatic shear banding (ASB) during high strain-rate compression of AISI 1045 steel. Plane strain and cylindrical axisymmetric compressions are simulated in LS-DYNA, considering rectangular and cylindrical steel samples, respectively. Also, a parametric analysis in height-to-base ratio is conducted in order to evaluate the effect of geometry and dimensional ratio of the sample on ASB formation. Doubly structural-thermal-damage coupled finite element models are developed for the numerical simulations, implementing the thermo-viscoplastic Modified Johnson–Cook constitutive relation and damage criterion, while further damage-equivalent stress and strain fields are introduced for the damage coupling. The simulations revealed that plane strain compression promotes more ASB formation, providing lower critical strain for ASB initiation and wider and stronger ASBs compared with axisymmetric compression. Further, X-shaped ASBs initially form during plane strain compression, while as deformation increases, they transform into S-shaped ASBs in contrast to axisymmetric compression, where parabolic ASBs are developed. Also, a lower height-to-base ratio leads to greater ASB propensity, reducing critical strain in axisymmetric compression. Finally, thermal softening is found to precede damage softening and dominate the ASB genesis and its early evolution, while in contrast damage softening drives later ASB evolution and its transition to fracture.

## 1. Introduction

In high-speed metal forming, the plastic deformation goes through a high strain rate, which activates unstable deformation mechanisms strongly connected to dynamic failure. Their clear understanding allows not only for a proper design of the process regarding tool design and optimization, but also for preventing unstable deformation phenomena in order to minimize material defects and improve product quality. Adiabatic shear banding (ASB) represents such an unstable deformation mechanism which takes place under the high strain rate and reacts to uncontrollable strain evolution and rapid fracture. Its adiabatic character comes from the high strain rate, which does not allow enough time for heat diffusion. In that way, the internal heat, which is produced from the plastic work, is stored inside the deformed structure and causes a significant temperature rise under adiabatic conditions. In fact, the amount of plastic work that is converted to internal heat is described by the Taylor–Quinney coefficient, and it usually lies about 0.9, although Rittel et al. [1] recently reported that it depends on the material, strain level and loading state.

ASB has been described as thermomechanical shear strain instability and occurs under high strain and strain rates [2,3]. Specifically, ASB is initially manifested via severe shear localization as a deformed band (dASB), while the transformed band (tASB) is developed as strain progresses due to the significant temperature rise. That increase in temperature leads to material thermal softening while further activating phenomena like dynamic recrystallization (DRX) and phase transformation, which are considered two dominant mechanisms of tASB [2]. Following, the temperature rise within ASB generates hot spots, which evolve into micro-voids whose coalescence creates micro-cracks leading to dynamic fracture [2,3]. The cracking propagation develops along the ASB direction and is controlled by damage softening, which drives ASB evolution and fracture, in contrast to thermal softening, which has been found to appear earlier in determining ASB initiation [4].

Regarding ASB appearance in compressive loading, Tresca [5] first observed the occurrence of X-shaped shear bands during the forging process, describing them also as heat zones due to the massive temperature rise coming from the intense shear localization. In many circumstances, ASBs follow the traces of slip lines during compressing deformation, affecting the velocity flow field and the compressing force according to the Upper Bound Method [6]. Semiatin and Lahoti [7] examined Ti-6242 alloy subjected to isothermal hot forging and side pressing tests at different strain rates and initial temperatures. Workability maps were extracted, determining the safety forming limits with respect to strain rate and temperature, while an instability criterion was derived, introducing the ratio of work-hardening rate to strain rate sensitivity of flow stress. The above ratio was found to strongly affect the material propensity to ASB formation, revealing a critical value of 5 for ASB appearance. Wang et al. [8] examined AISI 201 austenitic stainless steel under multi-axial compression, showing that a 9 μm wide ASB generated about 54 μs after peak true flow stress, in contrast to the commonly accepted aspect which considers the timing of ASB genesis to coincide with the instability point. The temperature inside ASB reached 58% of the melting point, with hot spots being captured due to the non-uniform temperature distribution. Eventually, dynamic recovery and rotational dynamic recrystallization (RDR) dominated the microstructural evolution inside ASB. Xue and Gray [9] tested 316L stainless steel hat-shaped specimens under a dynamic compression of 2500 s^−1^ strain rate. The results indicated the work-hardening rate as a crucial material parameter for adiabatic shear localization, while the ASB initiation moment coincided with the peak shear stress point. Finally, the high defect density in dislocations and twins triggered shear localization and provided ASB with high hardness, while fracture along ASB was connected to the sudden stress collapse.

Special interest has also been focused on the investigation of ASB evolution in plane strain and uniaxial compression. Anand and Spitzig [10] studied the initiation of shear bands in plane strain compression of aged maraging steel. The orientation of shear bands was obtained at 55°, while the specimen was found to sustain enough deformation even after ASB and before fracture. Also, a theoretical expression of critical strain for shear localization was derived, however, a comparison between classical flow theory and experiments revealed deviations regarding ASB initiation. Harren et al. [11] studied the formation of shear bands in single crystals and polycrystals of Al-3%wt Cu alloy during plane strain compression, indicating that coarse slip precedes ASB and propagates through entire grains in the case of a single crystal and across grain boundaries regarding polycrystals following straight trajectories. Their work reported that damage softening is not prerequired for ASB genesis, while the last one occurs at an instability point or even slightly later. Finally, textural softening was considered the main cause of instability in polycrystals, as their slip systems kept on self-hardening with strain increase. In addition, Prakash et al. [12] experimentally and numerically investigated the formation of macroscopic shear bands (MSBs) of aluminum alloys subjected to plane strain compression at 298 and 573 K temperatures. MSBs were detected for Al-6%wt Mg alloy at 573 K, forming at 20% strain along the diagonals of the cross-sectional area, while lower series of aluminum alloys did not significantly form MSBs. MSB microstructure was characterized by high hardness and misorientation, together with grain elongation and fragmentation. At last, the simulations predicted an earlier MSB onset revealing lower critical strain. Moreover, Tang et al. [13] examined the ASB formation in Inconel 718 nickel-base superalloy during plane strain and uniaxial compression. The results indicated a more intense ASB evolution in plane strain deformation, while X-shaped and S-shaped ASBs were detected regarding their traces. The X-shaped bands formed under mild deformation conditions with lower strain rate and higher temperature, in contrast to S-shaped bands which developed under more severe conditions of higher strain rate and cold working. Finally, a distinction between branching ASBs was made by defining stronger and weaker bands.

Regarding ASB development in uniaxial compression of cylindrical specimens, Odeshi and Bassim [14] studied quench-hardened and tempered AISI 4340 steels at different tempering temperatures. The role of adiabatic heating and thermal softening was proved dominant in driving the unstable deformation, leading to parabolic-shaped ASBs. An S-shaped fracture path was developed along the trace of ASB, while ductile, shearing and knobby fracture modes were detected in all tempering temperatures. In fact, the knobby feature of the fractured structure revealed the existence of local melting inside ASB, generating void nucleation whose growth also provided dimpled fracture. Similarly, Odeshi et al. [15] reported two parabolic cones of martensitic transformed ASBs mirroring each other and forming during high strain rate compression of AISI 4340 steel cylinder. The trace of ASBs looked circular or elliptical through the transverse section and parabolic through the longitudinal one. The adiabatic heating and thermal softening generated micropores whose coalescence led to cracking propagating along ASB at about 45°. Dual-phase and martensitic steel cylinders have also been examined against dynamic impact [16], revealing a higher resistance to ASB formation for martensitic steel due to more homogeneous deformation, while instead two parabolic ASBs appeared in dual-phase steel along two coaxial, symmetrical and hemispherical cells. Sun et al. [17] studied a cylindrical hexagonal close-packed (HCP) pure titanium sample, reporting a long diagonal ASB. The microstructure within ASB exhibited continuous DRX, while the grain orientation favored the prismatic slip in contrast to the basal slip system of the matrix. Finally, Zhang et al. [18] studied Al-4.2%wt Cu cylinders under electromagnetic impact, reporting 135 μm wide ASBs which contained equiaxed, elongated and refined grains due to RDR. Both bands were parabolic, propagating at 34°, while the radial velocity gradient seemed to control their formation.

In general, a significant amount of research interest in the past has been concentrated on ASB formation in steels. In more detail, Lee et al. [19] examined SKS93 tool steel and S15C low carbon and S50C medium carbon steels under dynamic compression in a split Hopkinson pressure bar (SHPB). The results indicated that increased carbon content provided higher narrower and hotter ASB with greater hardness, while increased strain rate resulted in lower ASB width, higher hardness and temperature. Low-carbon steel developed only dASB, in contrast to medium and high-carbon steels, which formed both dASB and martensitic tASB. Finally, the fracture surface of SKS93 and S50C steels consisted of a dimple-like structure with knobby features whose size increased with strain rate and carbon content. Kang et al. [20] also studied different carbon steels (0.2–0.3–0.4–0.8%wt C) subjected to cold forging at SHPB, concluding that higher pearlite volume fraction leads to easier ASB formation. In all cases, the diagonal ASB that was formed contained ferrite and pearlite, except the 0.8%wt C steel in which only a pearlitic ASB microstructure was detected. In that case, a white-colored tASB was developed with 45° deep cracking. Nakkalil [21] studied eutectoid steels under high strain rate compression at different temperatures, revealing that alloy steels and lower temperatures favor shear localization. Also, discontinuous load drops were connected to the formation of tASB, while a higher strain rate seemed to decrease critical strain for ASB initiation. At last, the propensity to form tASB was found to increase under higher strength, higher hardness and finer pearlite interlamellar spacing. In addition, Syn et al. [22] studied pearlitic 1.3% C steel, reporting a 44 μm wide ASB which initiated at 55% strain. Austenitic phase transformation was detected due to adiabatic heating, as well as retransformation through rapid cooling, providing high hardness to the ASB core. Li et al. [23] conducted dynamic compression tests in high Co-Ni M54 steel, obtaining diagonal ASBs that formed at 23% strain under both deformed and transformed types while shearing failure was manifested via 45° cracking at 28% strain. The tASBs were found to be narrower and contained fine equiaxed nanograins caused by RDR. Finally, Jo et al. [24] investigated high-strength 500T armor steel under 3900 s^−1^ compression tests at SHPB, revealing that dASB initially formed right after stress collapse and next converted to tASB, leading to cracking failure. DRX equiaxed nanograins were observed, while RDR mechanism and grain-growth rate models were developed based on temperature rise calculations.

Moreover, several microstructural factors have been found to affect the tendency of ASB development, like grain size and shape, grain rotation, texture, alloying elements and twinning orientation [25,26]. In fact, twinning orientation has been considered a triggering feature for DRX at high temperatures, while prior heat treatment, initial temperature and strain rate affect grain size and ASB susceptibility in consequence [25]. Further, ASB seems to widen as strain increases, and elongated DRX grains enlarge at increased deformation temperature [27]. In more detail, DRX was proposed as the dominant softening mechanism leading to ASB failure, instead of thermal softening [28,29]. DRX was found to precede ASB genesis and destabilize the deformation by weakening the material through dislocation-free nanograins [28]. Therefore, DRX can be described more as a precursor rather than an outcome of ASB [28,29]. Oppositely, Guan et al. [30] suggested dynamic recovery as the dominant mechanism for grain refinement in Ti-1023 alloy instead of DRX. The grain refinement inside ASB was separated into discrete stages, while several phase transformations were detected together with the refined grains. Finally, Muiruri et al. [31] examined the ASB tendency on additively manufactured Ti-6Al-4V alloy, concluding that both as-built (AB) and stress-relieved (SR) specimens exhibited ASB during dynamic compression, with a more ductile fracture via equiaxed voids, cracks and elongated parabolic dimples in the case of SR samples.

This work studies the development of ASB in the high strain-rate compression of AISI 1045 steel, focusing on the investigation of ASB formation during plane strain and axisymmetric uniaxial compression. Numerical simulations were conducted in LS-DYNA software by developing a doubly structural-thermal-damage coupled finite element (FE) modeling approach, which considers a thermo-viscoplastic material model for plasticity flow rule and a thermo-viscoplastic damage criterion. For both material and damage models, the Modified Johnson–Cook formula was implemented. The parallel and conjugated deployment of structural-thermal and structural-damage couplings allows for evaluating the effect of thermal and damage softening mechanisms on ASB evolution. Thus, the double coupling permits the analysis of the interaction and the competition of the two softening mechanisms, attributing distinct roles regarding the initiation and propagation of ASB, as well as its transition to fracture. The current study compares the ASB formation during plane strain and axisymmetric high strain rate compression, aiming to investigate the effect of loading state and geometry (rectangular/cylindrical) on adiabatic shear instability. In addition, a parametric analysis of the height-to-base ratio was further carried out in order to investigate its influence on ASB trace and initiation, respectively. For each case, this analysis was focused on evaluating the ASB shape, orientation, trajectory and width by visualizing the strain and temperature fields, while the magnitude of the damage field along ASB also allowed the assessment of its influence on driving ASB evolution compared to the temperature profile. Finally, the transition from ASB to fracture was also examined, aiming to capture the cause of cracking genesis and evaluate the damage localization and hot spots that lead to voiding generation.

## 2. Methodology

### 2.1. Examined Configuration

**Figure 1:**
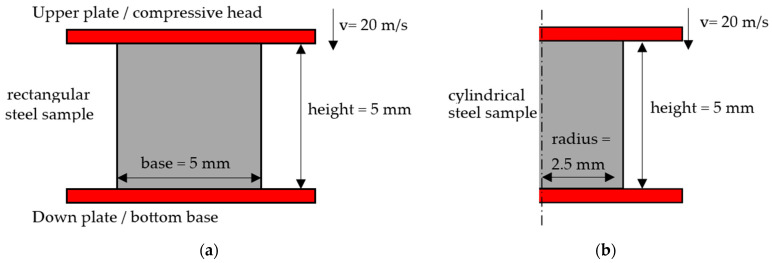
Examined configurations: (**a**) plane strain compression model; (**b**) cylindrical axisymmetric compression model.

This work studies the formation of ASBs during dynamic compression of AISI 1045 steel samples under plane strain and axisymmetric loading. Specifically, plane strain compression examines rectangular steel samples, while axisymmetric uniaxial compression refers to cylindrical steel samples. In both cases, 2D analysis is conducted on the cross-section of the samples, while in the case of cylindrical axisymmetric compression, the half domain is studied due to the cylindrical axisymmetric geometry. Figure 1 depicts the basic examined configurations for the plane strain and axisymmetric compression, showing that both samples were 5 mm in height as well as in their base. The compressing velocity was considered constant at 20 m/s, revealing an initial strain rate of 4000 s^−1^ for the compression, which allowed for adiabatic loading conditions. In addition, both upper and down plates were treated as rigid and undeformable steel bodies of high enough width in order to retain contact with the specimen during the deformation. Finally, a maximum termination time of 200 μs was adjusted, allowing for high enough strain levels in order to secure ASB formation and its transition to fracture.

### 2.2. FE Modeling

#### 2.2.1. Mesh Generation

In this study, FE simulations were carried out in LS-DYNA software (Livermore Software Technology Corporation, Livermore, CA, USA) in order to numerically investigate the ASB formation in plane strain and cylindrical axisymmetric dynamic compression of AISI 1045 steel. The FE models were developed in the LS-PrePost 2024R2(4.11) tool, where the post-processing of the results also took place. In both cases of the plane strain and axisymmetric compression models, 2D analysis was carried out by simulating the problem alongside the sample specimen, while in the case of axisymmetric problem, the half-axisymmetric domain was studied.

In more detail, the FE mesh consisted of quadrilateral 4-node 2D solid elements with an initial mesh size of 10 μm regarding the steel specimen, while a sparser mesh was applied to the upper and down plate, which were considered rigid bodies through the *Mat020_rigid* keyword. In fact, the mesh size of the specimen was selected low enough in order to sufficiently capture the ASB width. The meaning of 2D solid elements refers to the condition that the computations of stiffness and mass matrices consider solid elements, but under a 2D form, due to the 2D space of the simulated problem. More specifically, the element type was selected via the *ELFORM* parameter for 2D plane strain and 2D axisymmetric solid elements separately for each simulation case. Also, a Lagrangian mesh type was applied through the *SETYP* parameter. In fact, the adjustment of Lagrangian mesh type was made due to the fact that it connects the mesh nodes to the material and enforces them to move together with material deformation, allowing in that way for the detection of the plastic flow trace through grid trajectories.

Further, the r-adaptive remeshing technique was implemented in order to face the massive mesh distortion due to severe shear deformation, decreasing the simulation time and improving the computational accuracy. In particular, r-adaptive re-meshing readjusts the mesh nodes within the entire domain without adding new degrees of freedom, while the nodal readjustment strategy aims to retain the product of element size and computational error to be relatively constant. In that way, the mesh is refined in areas of higher errors, allowing for greater accuracy and preventing computational instabilities, which can lead to a negative Jacobian determinant due to severe shear deformation. Eventually, the adaptive timestep plays an important role during mesh adaptation as it must be low enough to capture the rapid evolution of ASB but also to allow for a smoother mesh re-generation, which can face multiple challenges due to negative element volume coming from element erosion due to material failure.

Finally, the Flanagan–Belytschko stiffness formula was implemented for hourglass control. That hourglass control prevents elements from experiencing hourglass deformation during strain evolution, which leads to computational instabilities and zero-energy deformation modes during numerical solution due to volumetric blockage [32].

#### 2.2.2. Boundary Conditions

The boundary conditions were implemented in order to prevent the interacting contacts from penetrating. For this reason, two contact algorithms were activated, which were the *2D_Automatic_Surface-to-Surface* and the *2D_Automatic_Single Surface* algorithms. In particular, the 2D surface-to-surface algorithm was implemented for the interacting interfaces in the contacts between the steel sample and the plates. In both cases, the steel sample was considered the deformable body and the plates the rigid ones. On the other hand, the 2D single-surface algorithm was applied to the steel specimen in order to prevent self-penetration between the interacting cracking edges, which was generated from the damage evolution at high strain, and the additional compressive load tended to bring them in contact. Both contact algorithms are described as 2D due to the fact that they acted in both the simulated plane and its perpendicular one. Further, both contact algorithms are two-way algorithms, meaning that they check the penetration between the nodes of the one body and the interacting surface, as well as the opposite case. In order to avoid penetration, both contact types introduced a penalty force to the penetrating nodes, which represented the contact reaction force.

#### 2.2.3. Material and Damage Modeling

In the current work, a doubly coupled FE analysis was carried out by implementing a structural-thermal-damage coupling during modeling development. In more detail, the two couplings took place between structural-thermal and structural-damage fields, allowing for simulation of the effect of thermal and damage softening mechanisms, respectively.

For the material modeling of AISI 1045 steel, the Modified Johnson–Cook (MJC) flow rule was implemented, which considered a thermo-viscoplastic constitutive relation for material plasticity. In that way, the MJC formula proposed that plastic flow stress depends on strain evolution, strain rate and material temperature, considering the effects of strain hardening, strain rate sensitivity and thermal softening, respectively. Equation (1) describes the MJC constitutive relation for plastic flow stress *σ*, showing that it is composed of three parts: the strain hardening term, the strain rate hardening term and the thermal softening term. *A*, *B*, *C*, *n* and *m* are the MJC material parameters, while r and $\dot{r}$ refer to the damage-equivalent strain and strain rate, respectively. The non-dimensional homologous temperature $T^{*}$ is calculated through material temperature *T*, room temperature *T_r_* and melting point *T_m_* as Equation (2) describes, while Equation (3) defines the normalized damage-equivalent strain rate ${\dot{r}}^{*}$ by correcting its real magnitude to the reference strain rate ${\dot{\varepsilon }}_{0}$. In fact, the strain rate hardening term in the MJC constitutive relation is activated for strain rates greater than ${\dot{\varepsilon }}_{0}$ value.

(1) $$\sigma =(A+B\cdot r^{n})\cdot {\left(1+{\dot{r}}^{*}\right)}^{C}\cdot (1-{T^{*}}^{m})$$

(2) $$T^{*}=\frac{T-T_{r}}{T_{m}-T_{r}}$$

(3) $${\dot{r}}^{*}=\frac{\dot{r}}{{\dot{\varepsilon }}_{0}}$$

The MJC formula differs from the classical Johnson–Cook material model by the fact that it considers a power-law strain rate hardening term instead of a logarithmic one while also accounting for the damage-equivalent strain and strain rate. The last ones are expressed in Equations (4) and (5), respectively, through the equivalent plastic strain ${\bar{\varepsilon }}^{p}$ and plastic strain rate ${\dot{\bar{\varepsilon }}}^{p}$. Specifically, the structural-damage coupled operation converts the strain and strain rate fields to the damage-equivalent ones through the damage coupling parameter *β* and damage parameter *D*. In this work, a fully damage-coupled analysis was carried out by adjusting the *β*-parameter equal to 1, although a zero value would allow for neglecting the damage softening effect and running a damage-uncoupled simulation under less computational cost.

(4) $$r={\bar{\varepsilon }}^{p}(1-\beta \cdot D)$$

(5) $$\dot{r}={\dot{\bar{\varepsilon }}}^{p}(1-\beta \cdot D)$$

Regarding damage modeling, *D*-parameter evaluates the damage magnitude reacting to fracture via element erosion when it reaches the value of critical damage *D_c_* which is equal to 1. For calculating the *D*-parameter, a thermo-viscoplastic MJC damage criterion is implemented, which considers the dependence of plastic strain failure *ε_f_* on stress field, strain rate and temperature. In more detail, Equation (6) describes the MJC damage law, which computes the plastic strain failure, where *D*_1_, *D*_2_, *D*_3_, *D*_4_ and *D*_5_ are the MJC damage parameters, ${\dot{\varepsilon }}^{*}$ the normalized strain rate and $\sigma^{*}$ the stress triaxiality. Specifically, Equation (7) defines the stress triaxiality as the ratio of hydrostatic pressure *σ_H_* to the equivalent Von Mises stress *σ_eq_*, while Equation (8) defines the normalized strain rate as the ratio of the equivalent plastic strain rate to its reference value ${\dot{\varepsilon }}_{0}$.

(6) $$\varepsilon_{f}=(D_{1}+D_{2}\cdot exp(D_{3}\sigma^{*}))\cdot {\left(1+{\dot{\varepsilon }}^{*}\right)}^{D_{4}}\cdot (1+D_{5}{Τ}^{*})$$

(7) $$\sigma^{*}=\frac{\sigma_{H}}{\sigma_{eq}}$$

(8) $${\dot{\varepsilon }}^{*}=\frac{{\dot{\bar{\varepsilon }}}^{p}}{{\dot{\varepsilon }}_{0}}$$

Therefore, the damage extent is assessed through the damage evolution Δ*D*, which represents the damage increment with time and accounts for the equivalent plastic strain increase $\Delta {\bar{\varepsilon }}^{p}$ with respect to plastic strain failure and critical damage, as shown in Equation (9). Further, an additional temperature-dependent damage criterion is applied via a critical temperature, which is set equal to the melting point. In that way, element erosion takes place when the material temperature reaches the critical temperature, simulating the local melting inside ASB, which results in micro-voiding [2,32].

(9) $$\Delta D=D_{c}\frac{\Delta {\bar{\varepsilon }}^{p}}{\varepsilon_{f}}$$

In addition, Equation (10) describes the damage-equivalent Von Mises stress ${\tilde{\sigma }}_{eq}$ relatively to the stress field and the damage extent. In that way, the structural-damage coupling conjugates the mechanical stress state with the damage evolution, simulating the damage-softening effect. In particular, with the increase in damage evolution, the greater *D*-parameter results in higher damage-equivalent stress together with lower damage-equivalent strain. In fact, the last one decreases the stress magnitude from Equation (1), reacting to increased stress triaxiality and, in consequence, a decrease in plastic strain failure due to the negative value of *D*_3_ damage parameter. Therefore, the influence of damage coupling on fracture initiation remains crucial as it reveals the effect of the damage softening mechanism.

(10) $${\tilde{\sigma }}_{eq}=\frac{\sigma_{eq}}{\left(1-\beta \cdot D\right)}$$

Equation (11) defines the plastic work rate ${\dot{W}}_{p}$, which is produced with deformation progress and is converted into internal heat under adiabatic loading at a high strain rate. The fraction of plastic work converted to internal adiabatic heat is described by the Taylor–Quinney coefficient *χ*, while the generated temperature reacts to significant temperature rise inside the deformed material. Equation (12) describes the temperature increase rate ∂T/∂t as an outcome of the generated heat $\dot{Q}$ which is equal to the product of *χ*-value with the plastic work rate, while *ρ* and *C_p_* refer to material density and specific heat, respectively. In fact, the heat conduction equation considers only the source term of heat generation, neglecting the heat diffusion term due to the high strain rate, which does not allow enough time for heat diffusion [2].

(11) $${\dot{W}}_{p}={\dot{r}\tilde{\sigma }}_{eq}={\dot{\bar{\varepsilon }}}^{p}\sigma_{eq}$$

(12) $$\frac{\partial T}{\partial t}=\frac{\dot{Q}}{\rho C_{p}}=\chi \frac{{\dot{W}}_{p}}{\rho C_{p}}$$

Table 1 contains the MJC material and damage parameters utilized in this study for AISI 1045 steel, together with its mechanical and thermal properties according to the literature data [33,34]. The MJC material and damage model parameters were introduced through *Mat107_Modified Johnson-Cook* material card in LS-DYNA. Finally, the strain rate parameters *C* and *D*_4_ of constitutive relation and damage law in Table 1 are corrected, considering that the classical Johnson–Cook accounts for a logarithmic strain rate hardening term, while in contrast, MJC considers a powered-law strain rate term.

#### 2.2.4. Computational Algorithm

**Figure 2:**
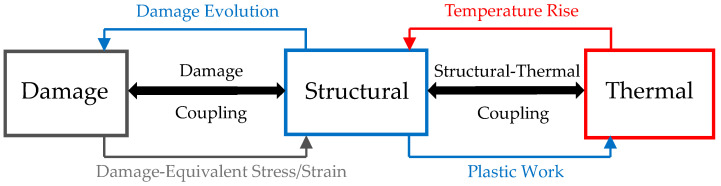
Structural-thermal-damage doubly coupled scheme.

In this study, the double structural-thermal-damage coupled FE analysis was developed by utilizing the explicit LS-DYNA code. The double coupling took place between structural-thermal and structural-damage coupled analyses. In particular, the structural-thermal coupled analysis allowed for simulating the effect of thermal softening on ASB formation, as the ASB strain instability exhibits when the magnitude of thermal softening overcomes the strain and strain rate hardening magnitudes. In other words, ASB macroscopic genesis coincides with the instability point of the stress–strain curve. Figure 2 illustrates that the structural solver feeds the thermal one with the produced plastic work of strain energy, which is converted to internal adiabatic heat via the Taylor–Quinney coefficient. That internal heat behaves like a source term for the thermal solver, which reveals the temperature field inside the deformed material by solving the heat conduction equation, neglecting, however, heat diffusion due to the high strain rate [2]. Next, the temperature rise is fed back to the structural solver which accounts for the effect of thermal softening on plastic flow stress. The interaction between the two solvers is feasible due to the introduction of the Taylor–Quinney coefficient and the implementation of a temperature-dependent constitutive relation like the MJC material model.

In addition, the coupling between the structural-damage fields allows for the assessment of the influence of the damage softening magnitude on ASB evolution and its transition to fracture. In fact, the double coupled analysis allows to investigate the influence of thermal and damage softening mechanisms on ASB initiation and evolution, attributing them distinct roles. Figure 2 shows that the structural solver calculates the damage evolution by assessing the damage magnitude and the plastic strain failure, while the damage part feeds back the structural solver with the damage-equivalent stress, strain and strain rate. The interaction between the two parts is feasible due to the implementation of a damage criterion and the introduction of the *β*-parameter, which is set equal to 1 for full coupling. The damage softening is predicted due to the fact that the damage-equivalent strain is lower than the original one for a certain damage extent, resulting in lower plastic flow stress, which increases stress triaxiality. Therefore, plastic strain failure decreases due to the negative *D*_3_ damage parameter, revealing fractures earlier and facilitating damage propagation.

**Figure 3:**
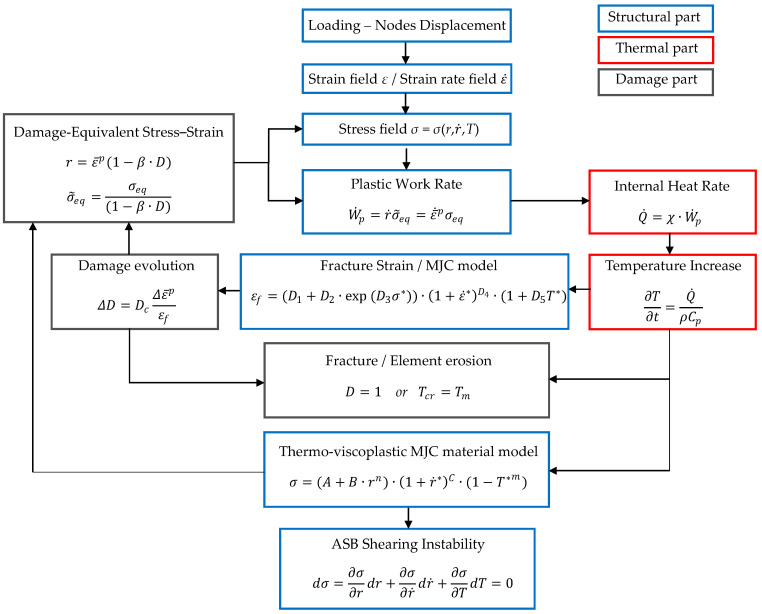
Flowchart of computational algorithm.

Figure 3 illustrates the schematic flowchart of the computational algorithm developed in this study. Specifically, the loading input was applied via the nodal displacement of the upper plate under the constant compressing velocity of 20 m/s. The nodal displacement, which was transferred to the steel specimen through the contact boundary condition of non-penetration, provided strain and strain rate fields, which resulted in the development of a stress state. The plastic strain energy was computed from the stress and strain fields and converted to internal heat via the Taylor–Quinney coefficient. Next, the increased temperature distribution was calculated from the heat conduction equation, neglecting, however, the heat diffusion term due to the high strain rate. That temperature increase was fed to the MJC formulas of constitutive relation and damage criterion from which the plastic flow stress and the plastic strain failure were assessed.

Following, the damage evolution was calculated, providing the damage-equivalent stress, strain and strain rate fields. The last ones were fed back to the MJC constitutive relation, allowing for the computation of plastic work for the next iteration. Before each iteration, the FE mesh was checked for possible element erosion representing the material failure. That element erosion takes place when the damage parameter *D* reaches the critical value of 1, or when the temperature reaches the critical value of melting point reacting to local melting within ASB and micro-voiding genesis. Finally, ASB initiation coincided with the stress instability point, and it was captured through the stress–strain curve, which provided the critical strain for ASB initiation. In that way, the ASB width was determined by obtaining the strain distribution transversely to the ASB direction and detecting the bandwidth of strain greater than the critical value.

## 3. Modeling Verification

The developed FE modeling approach was validated against experimental data provided from a high strain rate compression test of the AISI 4340 stainless steel cylindrical specimen. Specifically, the experiment which utilized during the validating procedure was conducted by Odeshi et al. [15], studying a 4340 steel sample of 9.55 mm in diameter and 10.55 mm in height. The dynamic compression test was carried out in an SHPB system under 1900 s^−1^ strain rate, while the stress–time and stress–strain curves were produced. In addition, the ASB formation was observed through optical micrographs along the longitudinal section of the impacted sample.

For the validating purpose, a similar FE model was developed for the same tested geometry and loading conditions of the 4340 steel cylindrical sample. The compressing velocity was adjusted properly in order to match the strain rate of the test, while the MJC material and damage parameters for the 4340 steel were selected according to the literature data [35], as Table 2 summarizes. In fact, the modeling validation was carried out by selecting experimental data from the dynamic compression test for 4340 steel, despite the fact that this work studies the ASB formation in high strain rate-impacted 1045 steel. The difference between the studied material and the one utilized to verify the numerical accuracy of the models is attributed to the lack of sufficient experimental data for high strain rate-compressed 1045 steel, given that the FE models only macroscopically and mechanically analyze the ASB formation instead of microstructurally, in which the majority of the tests often focus.

**Figure 4:**
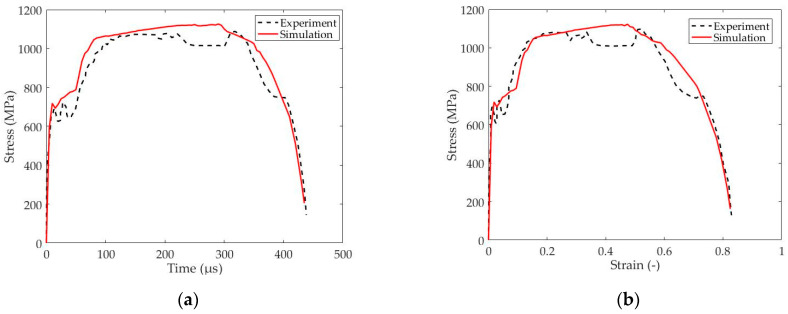
Comparison in stress fluctuation during deformation: (**a**) stress–time curve; (**b**) stress–strain curve (Experiment–Odeshi et al. 2005, [15]).

**Figure 5:**
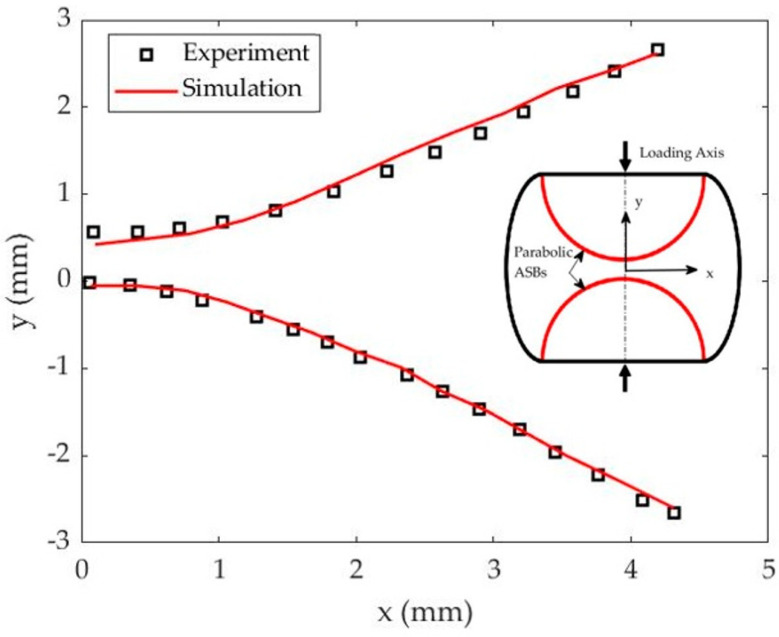
ASB trajectories through the longitudinal section in simulation and experiment (Experiment–Odeshi et al. 2005, [15]).

Therefore, the comparison between the model and the experiment is focused on the stress–time and stress–strain curves which reveal the timing *t_cr_* and the critical strain *ε_cr_* for ASB initiation which coincides with the instability point of peak stress, while the accuracy in predicting the ASB trajectory is also evaluated. In particular, Figure 4 depicts the stress fluctuation with time and strain, showing that the prediction of flow stress by the simulation comes in sufficient agreement with the experimental one. Specifically, the simulation predicted a peak stress of 1123 MPa, revealing a relative error of about 2%, while the instability point was predicted to exhibit a critical strain of 0.486 at 297 μs providing deviation against the experiment below 7%, as Table 3 describes. Finally, both simulation and experiment revealed two conical-shaped and parabolic ASBs which formed along the maximum shear direction and met at the sample center, while Figure 5 illustrates the ASB trajectories in the half-axisymmetric domain of the longitudinal section, indicating that the trace of upper and lower parabolic ASBs was captured accurately by the FE simulation.

## 4. Results and Discussion

### 4.1. Simulation Cases

This work numerically studies the ASB formation during dynamic plane strain and axisymmetric compression of AISI 1045 steel. The developed models implemented a doubly coupled FE method by carrying out structural-thermal-damage coupled analyses in order to evaluate the effect of thermal and damage softening mechanisms during ASB evolution.

The case studies of the numerical simulations in LS-DYNA are divided into two main parts. First, the ASB development during plane strain and cylindrical axisymmetric compression problems was studied in order to indicate the influence of each loading state and its propensity to ASB formation and evolution. Specifically, a rectangular steel sample was considered for the plane strain compression problem, while a cylindrical steel one was considered for the uniaxial axisymmetric problem. In both cases, the dimensions of the steel samples were the same, with both the base and the height being equal to 5 mm, while both compression problems were simulated at room temperature.

Secondly, a parametric analysis of the height-to-base ratio was conducted for both plane strain and uniaxial axisymmetric compression problems, aiming to identify the influence of the sample dimensional ratio on the ASB formation. In particular, the effects on ASB trajectory, width and intensity were studied, as well as the timing of ASB genesis. The examined values of the height-to-base ratio were 0.75–1–1.25, while in all cases, the height of the steel sample was maintained at 5 mm. Therefore, the sample base was the dimension, which varied in each case in order to match the desired height-to-base ratio. In the case of the rectangular steel samples under plane strain compression, the base referred to the specimen width, while in the case of axisymmetric compression, the base referred to the diameter of the cylindrical steel sample. The selection of a common initial height aimed to provide the same initial strain rate of 4000 s^−1^ for all case studies as a constant 20 m/s compressing velocity was applied in all simulation cases. Finally, Table 4 demonstrates a description of the developed models in this work, while Table 5 summarizes the comparisons between the specific models for each simulation case study.

### 4.2. Effect of Strain Field Problem

**Figure 6:**
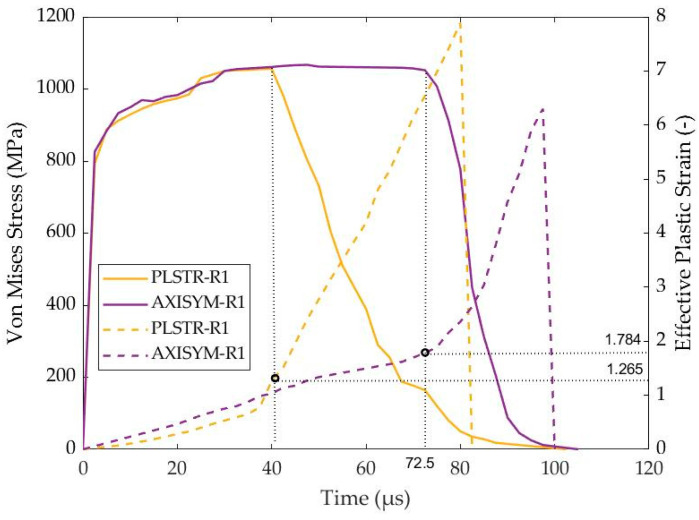
Flow stress (solid lines) and effective plastic strain (dashed lines) with time for PLSTR-R1 and AXISYM-R1 simulations.

This case study investigates the ASB development during high strain rate compression of 1045 steel under plane strain and axisymmetric loading states. In the first case, a rectangular sample geometry of 5 × 5 mm was considered via the PLSTR-R1 model, while in the second case a cylindrical sample with the same dimensions was simulated through the AXISYM-R1 model. Figure 6 illustrates the time fluctuation of the flow stress and the effective plastic strain inside the shear localization zone (SLZ) for the two models showing that plane strain loading condition accelerates ASB formation, reacting to fracture earlier compared with cylindrical axisymmetric compression. From a macroscopic point of view, ASB genesis coincided with the strain instability point of peak stress. Therefore, the PLSTR-R1 model predicted that ASB would be generated at 40 μs revealing a critical effective plastic strain of 1.265 for ASB initiation, while in contrast the AXISYM-R1 model estimated ASB occurrence quite later at 72.5 μs providing a critical strain of 1.784. In fact, the critical strain value referred to the magnitude of effective plastic strain inside SLZ, above from which ASB was generated allowing for distinguishing ASB from SLZ. Similarly, considering the constant velocity of 20 m/s and the initial height of 5 mm, ASB exhibited at 17.4% and 34% compressive true strains for plane strain and axisymmetric compression, respectively, declaring also that plane strain loading facilitated ASB formation. Thus, the earlier ASB initiation at plane strain compression resulted in a sooner fracture, while the intensity of the ASB core was also greater due to higher peak localized strain reaching 8 in contrast to axisymmetric compression, in which peak strain within ASB rose up to 6.3.

**Figure 7:**
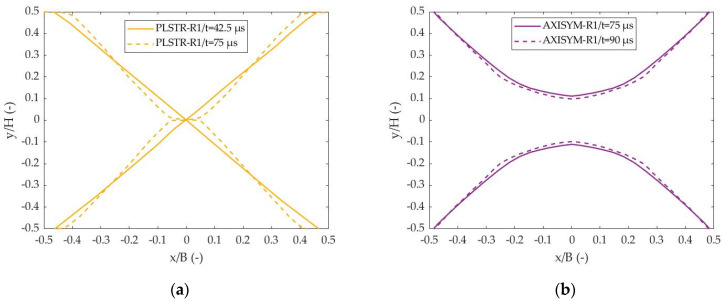
ASB trajectories at different times: (**a**) plane strain compression (PLSTR-R1 model) at 42.5 and 75 μs; (**b**) axisymmetric compression (AXISYM-R1 model) at 75 and 90 μs.

Figure 7 depicts the trajectories of ASBs at two different times, one slightly after their formation and one at their final stage. The non-dimensional cartesian coordinates x/B and y/H were utilized with B and H referring to the width and the height of the steel sample, while the origin was positioned in the center of the sample. In more detail, Figure 7a shows that ASBs were initially manifested as X-shaped bands at plane strain compression, while the central kink, which acted as their meeting point, was elongated as deformation increased, providing S-shaped ASBs at the final stage. On the other hand, cylindrical axisymmetric compression provided two parabolic ASBs, which maintained their shape during deformation progress, as Figure 7b depicts.

**Figure 8:**
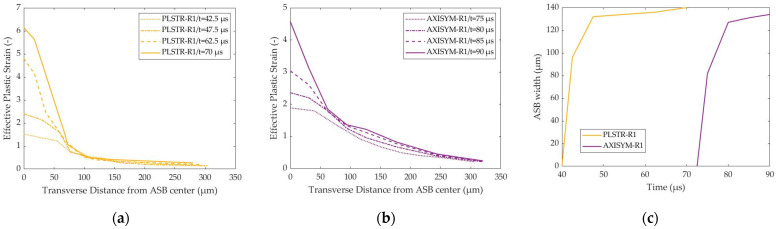
Transverse distribution of effective plastic strain and ASB width evolution with time: (**a**) strain transverse distribution with time for PLSTR-R1 model; (**b**) strain transverse distribution with time for AXISYM-R1 model; (**c**) ASB width evolution with time.

Further, Figure 8a,b depict the distribution of effective plastic strain transversely to ASB direction for plane strain and axisymmetric compression, respectively, allowing for the detection of ASB width as the extent of the area with higher strain than the critical value. Thus, the zone of strain higher than 1.265 and the one with strain higher than 1.784 predicted the semi-width of the ASBs at various times in the case of the plane strain and axisymmetric compressions, respectively, while Figure 8c shows the ASB width evolution with time. The earlier ASB initiation in the case of the PLSTR-R1 simulation revealed greater strain magnitude inside the ASB core, while in both cases the ASBs widened rapidly at first and then they seemed to reduce their widening rate as their transition to fracture began. Figure 8c shows that ASB width in plane strain compression was marginally greater, reaching about 140 μm in contrast to the axisymmetric case where ASB widened until it reached 134 μm.

**Figure 9:**
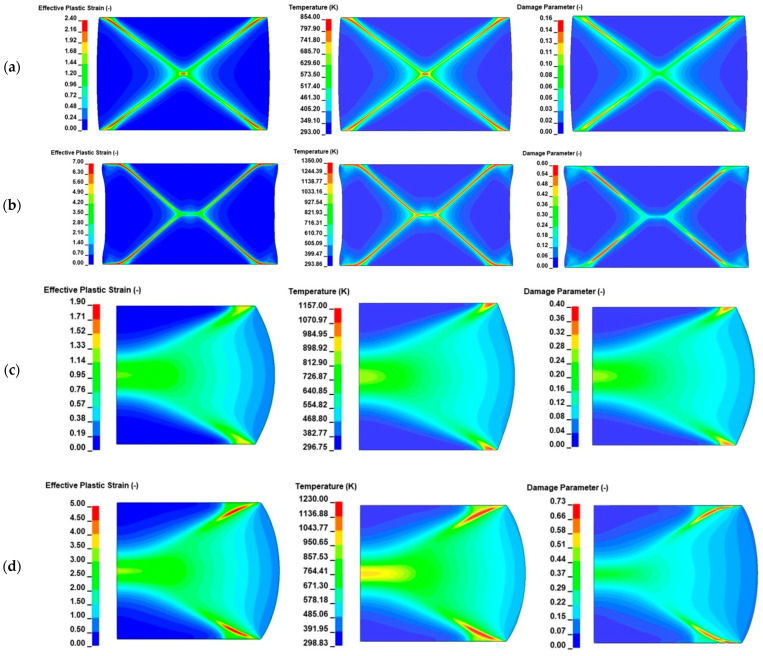
Effective plastic strain, temperature and damage fields for plane strain (PLSTR-R1) and axisymmetric (AXISYM-R1) compression at different times: (**a**) PLSTR-R1 model at 47.5 μs; (**b**) PLSTR-R1 model at 75 μs; (**c**) AXISYM-R1 model at 75 μs; (**d**) AXISYM-R1 model at 92 μs.

Figure 9 visualizes the strain, temperature and damage fields at different times for both examined cases. Specifically, Figure 9a depicts that X-shaped ASBs were initially formed at plane strain compression with higher intensity around the sample corners and propagated along the maximum shear direction until the central kink. In fact, the temperature rise seemed significant at the sample corners, triggering ASB generation there, while damage magnitude seemed more intense moving slightly sideways, driving the ASB evolution until it reached the center. At a higher compressive strain, S-shaped ASBs seemed to form by horizontally elongating the central meeting point, which attributed to the ASB’s trajectory in an S-like trace, as depicted in Figure 9b. On the contrary, the respective fields seemed more diffused in the case of axisymmetric compression as shown in Figure 9c,d, where the distinct detection of the ASB trace seemed more challenging. In fact, strain and damage tended to be more localized as deformation increased in contrast to temperature field, which seemed more diffused, providing a wider heat-affected zone.

**Figure 10:**
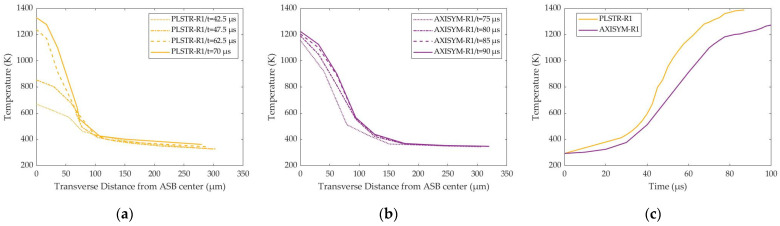
Transverse distribution and time fluctuation of temperature: (**a**) PLSTR-R1 model; (**b**) AXISYM-R1 model; (**c**) temperature evolution with time.

In addition, the temperature rise was more intense in the case of plane strain compression, while its variance with time also seemed greater, as confirmed in Figure 10. On the other hand, the cylindrical sample revealed a decreased temperature increase rate and lower peak temperature, but it showed a more stabilized transverse temperature distribution with time. However, the last one can be attributed to the smaller time steps, which were recorded as ASB generated later in the case of axisymmetric compression, and thus its evolution time until fracture was smaller, while much later, ASB appearance led to higher initial temperatures inside its core compared with the plane strain compression simulation.

**Figure 11:**
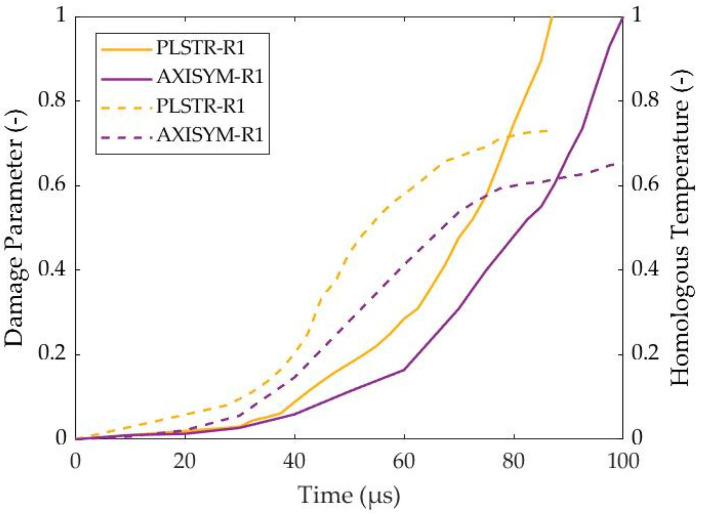
Evolution of damage (solid lines) and homologous temperature (dashed lines) with time.

In this work, the damage parameter reflected proportionally the magnitude of damage softening during the fully coupled structural-damage analysis under a *β*-parameter equal to 1, and the homologous temperature also reflected proportionally the magnitude of thermal softening due to the *m*-parameter, which is equal to 1 in the MJC constitutive relation. In that way, Figure 11 reveals that thermal softening mechanism is responsible for ASB genesis and its initial evolution due to its stronger magnitude at earlier stages around 40 μs and 72.5 μs for plane strain and axisymmetric compressions, respectively. On the other hand, damage softening seems to drive the later ASB evolution stages and its transition to fracture when the damage parameter overcomes the magnitude of homologous temperature.

**Figure 12:**
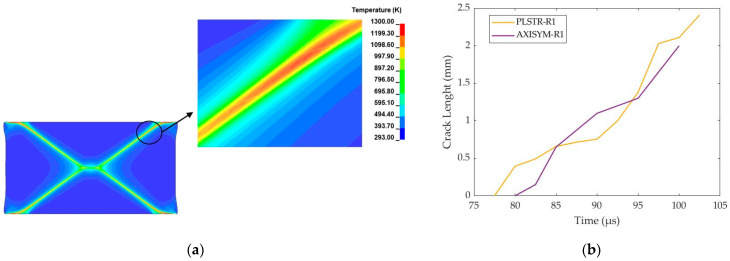

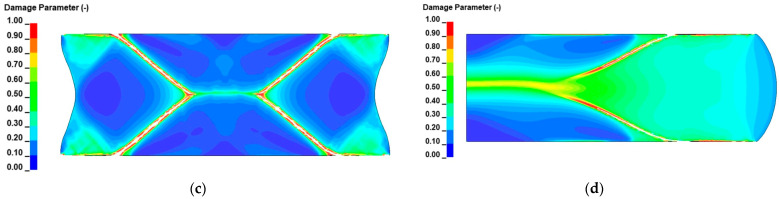
Hot spots inside ASB and cracking length with time: (**a**) hot spots inside ASB core in plane strain compression; (**b**) cracking length with time; (**c**) cracking development within damage field for PLSTR-R1 simulation; (**d**) cracking development within damage field for AXISYM-R1 simulation.

Specifically, the transition from ASB to fracture was enhanced by the increased damage extent and dispersed hot spots which acted like fracture triggers. In fact, the hot spots shown in Figure 12a, together with damage increment, generate micro-voiding, which was elongated along the ASB direction, creating micro-cracks whose coalescence resulted in cracking failure via mode-II cracking propagating. Figure 12b depicts the evolution of cracking length with time, showing that the mean propagation velocity of cracks was similar in both plane strain and axisymmetric compression cases. Finally, Figure 12c,d demonstrate that both rectangular and cylindrical samples fail under four developing cracks, which started from the corners and propagated along the softened ASB structure until the center of the sample and alongside the trace of the localized damage increment.

### 4.3. Effect of Height-to-Base Ratio

#### 4.3.1. Plane Strain Compression

**Figure 13:**
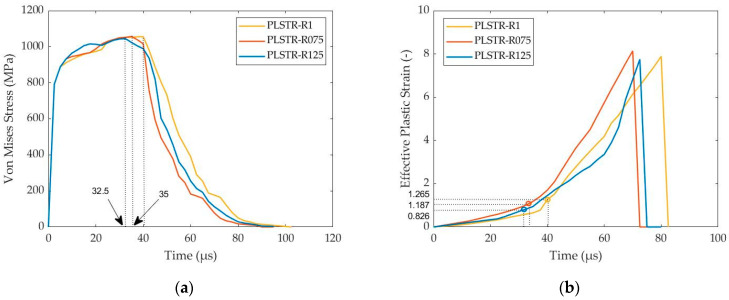
Flow stress and effective plastic strain variance with time for the plane strain models of different HBR: (**a**) flow stress–time curves; (**b**) effective plastic strain–time curves.

This case study investigates the influence of height-to-base ratio (HBR) on ASB formation during plane strain compression of orthogonal 1045 steel samples. The examined cases consist of 0.75–1–1.25 HBR values with the models named PLSTR-R075, PLSTR-R1 and PLSTR-R125, respectively. Figure 13a depicts the time fluctuation of flow stress, showing that ASB genesis appears at 35–40–32.5 μs and, in consequence, at compressive true strains of 15–17.4–14% for HBRs of 0.75–1–1.25, respectively. Looking at the time fluctuation of effective plastic strain in Figure 13b, the critical strain value to detect the ASB genesis lies at 1.187–1.265–0.826 for HBRs of 0.75–1–1.25, respectively. Therefore, the steel sample of HBR equal to 1.25 manifested earlier at the ASB onset, revealing the lowest critical strain, in contrast to the sample of HBR equal to 1, which delayed the ASB initiation more. In that way, rectangular geometries seem to increase ASB susceptibility compared with square samples, reacting to earlier ASB appearance. However, the ASB intensity seems almost equal for all models, reaching a peak effective plastic strain of about 8 inside its core.

**Figure 14:**
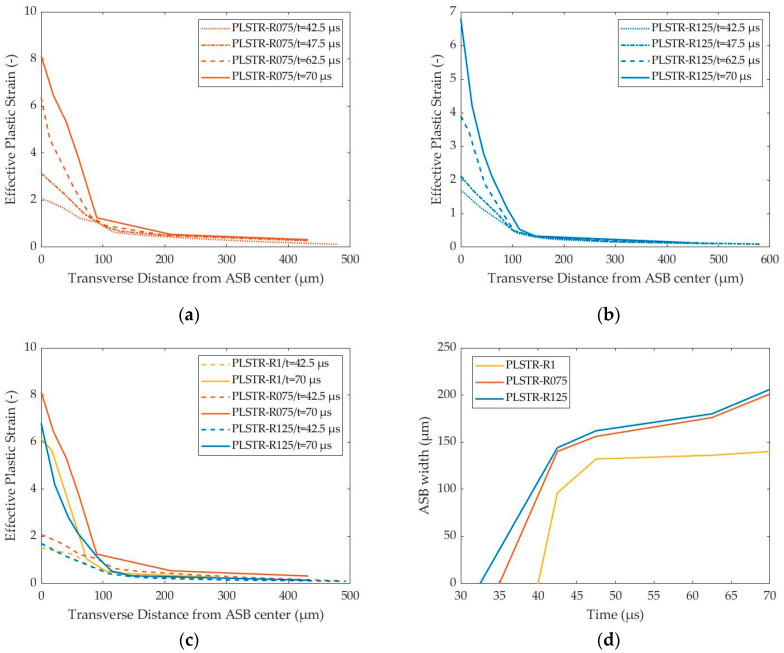
Transverse distribution of effective plastic strain and ASB width evolution with time for the plane strain models of different HBRs: (**a**) strain transverse distribution with time for PLSTR-R075 model; (**b**) strain transverse distribution with time for PLSTR-R125 model; (**c**) comparative strain transverse distributions at 42.5 and 70 μs; (**d**) ASB width evolution with time.

The earlier ASB generation allowed for more extensive strain fields transversely to the ASB direction without, however, significant difference in strain magnitude within the ASB core, as Figure 14a–c show. In more detail, ASB increased its interior strain localization and widened as deformation progressed, with the sample of HBR equal to 0.75 revealing a slightly higher peak strain, which reached up to 8. Also, Figure 14d demonstrates that rectangular samples revealed greater ASB width reaching up to 205 μm compared with the square sample (HBR = 1), which revealed a 140 μm wide ASB. The greater ASB width was attributed to the earlier initiation, which allowed it more time to expand transversely, and to the fact that ASB continued to widen even with a lower rate at higher deformation stages in contrast to the square sample where the ASB width seemed to be stabilized at final stages. In addition, the simulations with HBR different from 1 showed a different widening rate at first as their initial slopes were revealed, but they both showed a similar expanding rate afterwards with the PLSTR-R125 model reaching a slightly higher maximum width due to its earlier ASB onset.

**Figure 15:**
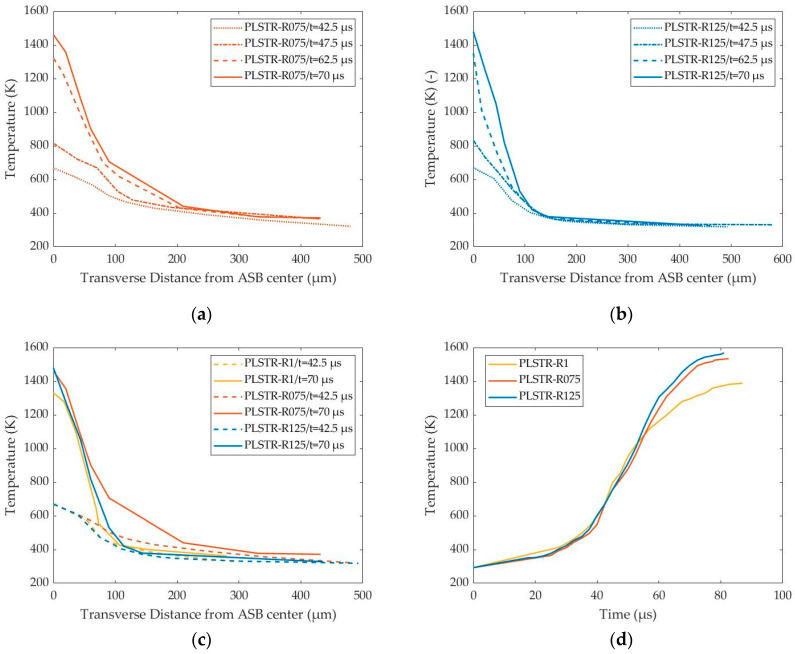
Transverse distribution of temperature with time and temperature-time fluctuation within ASB for the plane strain models of different HBR: (**a**) Temperature transverse distribution with time for PLSTR-R075 model; (**b**) Temperature transverse distribution with time for PLSTR-R125 model; (**c**) Comparative temperature transverse distributions at 42.5 and 70 μs; (**d**) Temperature–time curves inside ASB core.

Figure 15a,b illustrate the temperature distribution transversely to the ASB direction, showing that the heat-affected zone was wider than the actual ASB width due to the more diffused temperature field. Both PLSTR-R075 and PLSTR-R125 simulations reached a greater maximum temperature compared with PLSTR-R1, as confirmed in Figure 15c, due to their earlier ASB initiation, which allowed more time for ASB development during which the increased localized shear strain led to greater plastic work and internal heat, causing higher temperature rise. Also, Figure 15a,b demonstrate that the temperature field within ASB not only increased but also widened with time, showing a wider temperature localization in the ASB region compared with the strain localization, which was found to be narrower. Last but not least, Figure 15d reveals that the magnitude of temperature rise in each case of HBR value was almost the same until 55 μs, showing that the different geometries behaved similarly between each other during the stable plastic deformation and the early ASB stages. In fact, the early ASB stages around 40 μs revealed the greater temperature increase rate, as shown in Figure 15d, by the slope of the curves, while the final evolution stages of ASB seemed to reduce the temperature increase rate as their transition to fracture was dominated by the stronger damage softening mechanism.

**Figure 16:**
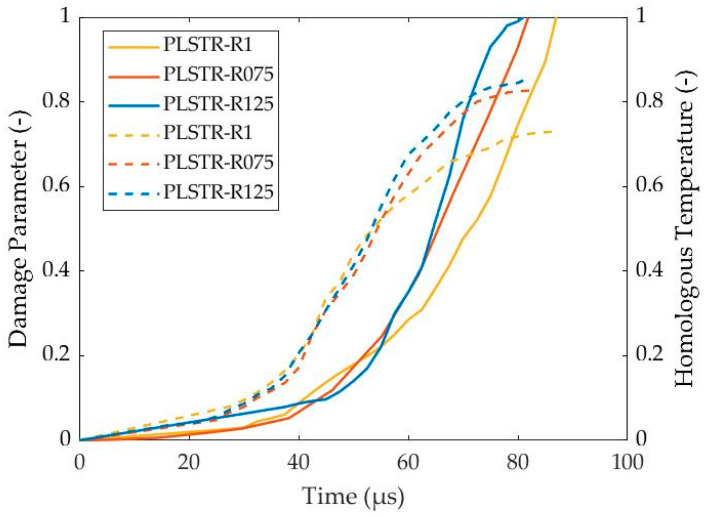
Evolution of damage (solid lines) and homologous temperature (dashed lines) with time for the plane strain compression models with different HBR values.

**Figure 17:**
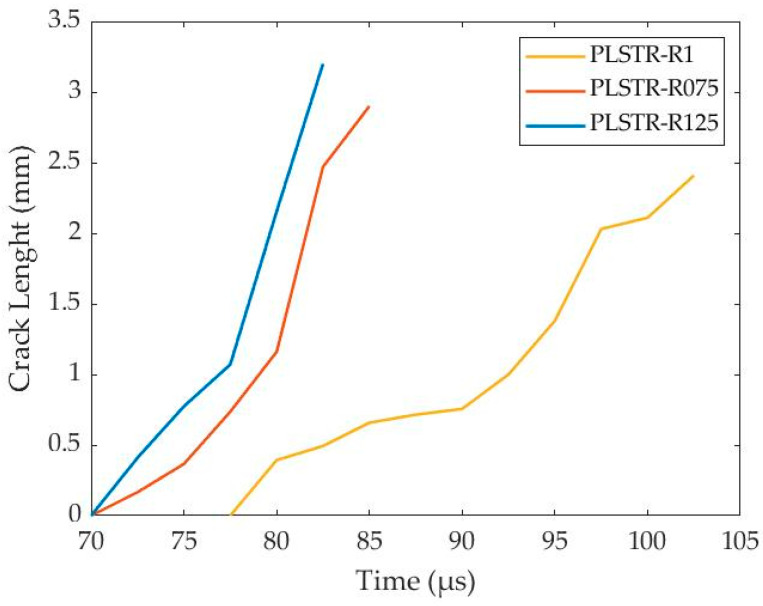
Cracking length with time for the plane strain compression models with different HBR values.

Furthermore, all simulations reveal that the significant temperature rise precedes the damage increment, as depicted in Figure 16. In that way, the thermal softening, which depends proportionally on the homologous temperature, exhibited earlier than the damage softening and was responsible for ASB genesis and its earlier development stages. In contrast, the damage softening became more superior at later stages, driving the final stages of ASB evolution and its transition to fracture, which took place when damage parameter reaches the critical value of 1. The fracture began via micro-voiding generation due to critical damage rather than local melting as the homologous temperature did not reach up to 1. As deformation progressed, the voids, which coincided with the positions of hot spots, elongated and created cracks through their coalescence. The cracks propagated along ASB direction as their trace consisted of softened structure due to significant temperature rise and damage extent, facilitating cracking propagation. Specifically, four cracks were generated initially around the corners, and propagated along the diagonals of the cross-section until the center of the sample, leading to the final failure. Finally, Figure 17 depicts the cracking length for each examined simulation, showing that the PLSTR-R075 and PLSTR-R125 simulations revealed greater cracking propagation velocity as obtained by their slopes due to their greater damage increase rate, as shown in Figure 16. Also, both models demonstrated earlier cracking initiation compared with the PLSTR-R1 model, while further, the cracks reached a slightly higher length compared with the case of the square steel sample of HBR equal to 1.

#### 4.3.2. Cylindrical Axisymmetric Compression

**Figure 18:**
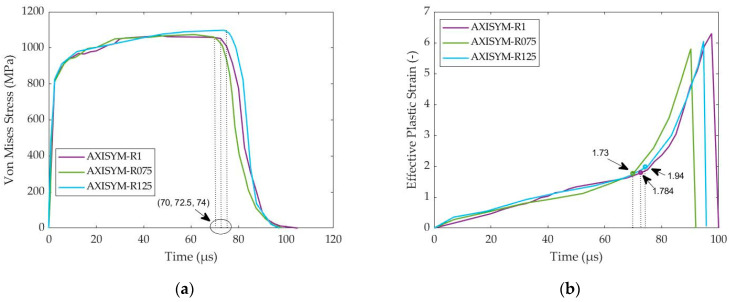
Flow stress and effective plastic strain variance with time for the axisymmetric models of different HBR: (**a**) flow stress–time curves; (**b**) effective plastic strain–time curves.

In this part, a similar parametric analysis of HBR was conducted regarding the axisymmetric compression of cylindrical 1045 steel samples. The examined values of HBR were 0.75–1–1.25 again, with the simulated models being AXISYM-R075, AXISYM-R1 and AXISYM-R125, respectively. As Figure 18a demonstrates, the AXISYM-R075 revealed the earliest instability point and, in consequence, the earliest ASB genesis at 70 μs and 32.9% compressive true strain considering the loading velocity of 20 m/s and the initial height of 5 mm, while AXISYM-R1 revealed the next ASB onset at 72.5 μs and 34% compressive true strain. On the other hand, AXISYM-R125 showed the greatest delay on ASB appearance, which took place at 74 μs and 35% true strain, concluding that lower the HBR was, the sooner the ASB was exhibited. Therefore, reduced HBR seems to promote ASB formation in cylindrical axisymmetric compression. With respect to the timing of ASB onset, the critical effective plastic strain values were detected for each case from Figure 18b, revealing the critical strains of 1.73–1.784–1.94 for the HBRs of 0.75–1–1.25, respectively. Also, the strain localization magnitude seemed to clearly increase with higher rate after ASB initiation, as obtained from the slopes of the curves in Figure 18b, while the earlier ASB initiation in the case of the AXISYM-R075 model led to sooner fracture.

**Figure 19:**
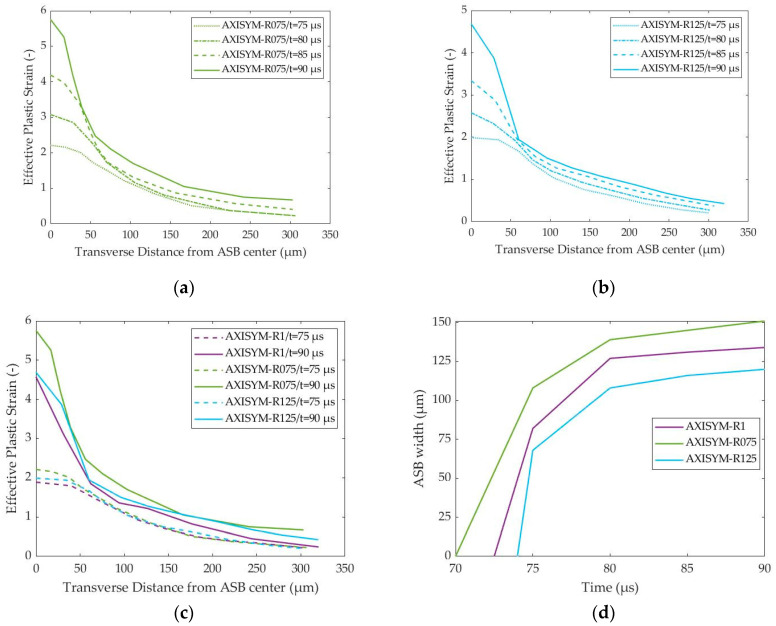
Transverse distribution of effective plastic strain and ASB width evolution with time for the axisymmetric models of different HBR: (**a**) strain transverse distribution with time for AXISYM-R075 model; (**b**) strain transverse distribution with time for AXISYM-R125 model; (**c**) comparative strain transverse distributions at 75 and 90 μs; (**d**) ASB width evolution with time.

Figure 19a,b show that the localized strain field inside ASB increased its magnitude and widened as deformation progressed for both HBRs of 0.75 and 1.25, as well as for the HBR of 1, as Figure 8b depicts. More specifically, the strain field within ASB reached higher peak strain with time increment and greater ASB width, which was determined as the zone extent characterized by effective plastic strain, which was higher than the critical strain. Figure 19c demonstrates that AXISYM-R075 revealed more intense strain localization with greater peak strain for the same time, which was attributed to the earlier ASB appearance which allowed more time for ASB to develop and grow. However, Figure 18b illustrates that the AXISYM-R1 and AXISYM-R125 models reveal higher final effective plastic strain inside the ASB core due to delayed fracture compared with AXISYM-R075. Finally, Figure 19d reveals that earlier ASB onset results in greater ASB width with AXISYM-R075 reaching a maximum width of 150 μm, which remained lower than the one in the case of plane strain compression, which lied about 205 μm. The above comes in agreement with the conclusion that plane strain compression promotes ASB formation more, leading to earlier initiation and greater width. Finally, all simulations showed a higher widening rate at the early stages of ASB evolution after its genesis, while the ASB width was almost stabilized at the final stages of ASB evolution before fracture.

**Figure 20:**
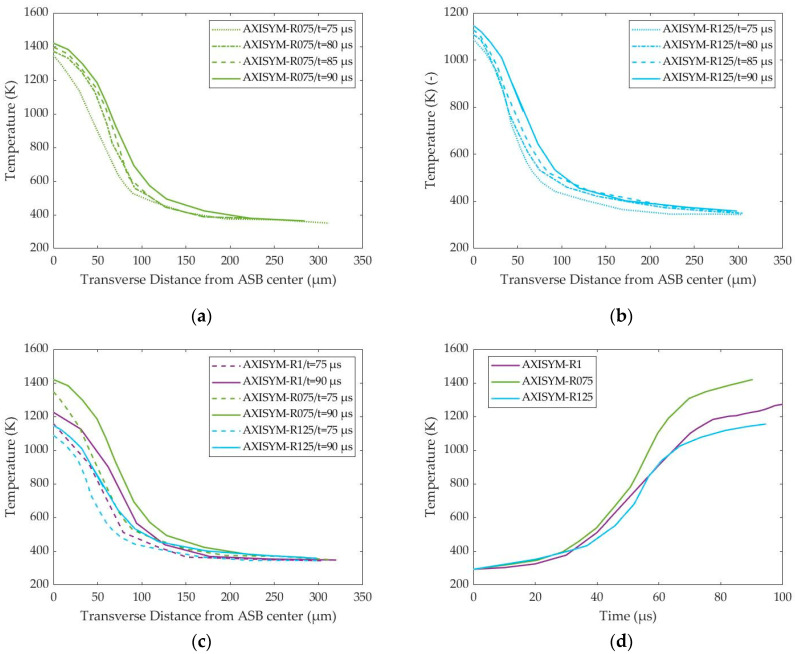
Transverse distribution of temperature with time and temperature–time fluctuation within ASB for the axisymmetric models of different HBR: (**a**) temperature transverse distribution with time for AXISYM-R075 model; (**b**) temperature transverse distribution with time for AXISYM-R125 model; (**c**) comparative temperature transverse distributions at 75 and 90 μs; (**d**) temperature–time curves inside ASB core.

In contrast to the transverse distribution of strain, temperature field seemed more diffused, revealing heat-affected zones that were wider than ASB. A significant temperature rise was obtained within the ASB core, softening the ASB structure thermally and facilitating damage propagation along the ASB direction. Figure 20a,b illustrate the temperature transverse distributions for axisymmetric models of HBR equal to 0.75 and 1.25, respectively, showing that the AXISYM-R075 model reached higher peak temperatures inside ASB due to its earlier ASB occurrence, which resulted in more developed ASBs. In that way, the higher peak strain, which reached up to 5.7 in Figure 19a, resulted in more severe shear strain localization and, in consequence, greater plastic work, which generated more internal heat, leading to a higher peak temperature inside ASB. This tendency is also captured in Figure 20c, which confirms that the sooner the ASB exhibits, the more intense the magnitudes of strain and temperature localization, revealing higher peaks. However, the heat-affected zones seem to possess a more constant width rather than widening with time as Figure 10b and Figure 20a,b depict. Finally, Figure 20d illustrates that greater temperature levels were achieved for a HBR of 0.75, which generated the ASB earlier, while the point where the temperature increase rate rose up seemed to precede the timing of ASB genesis, showing that heat generation acted more as a cause rather than an outcome of ASB formation.

**Figure 21:**
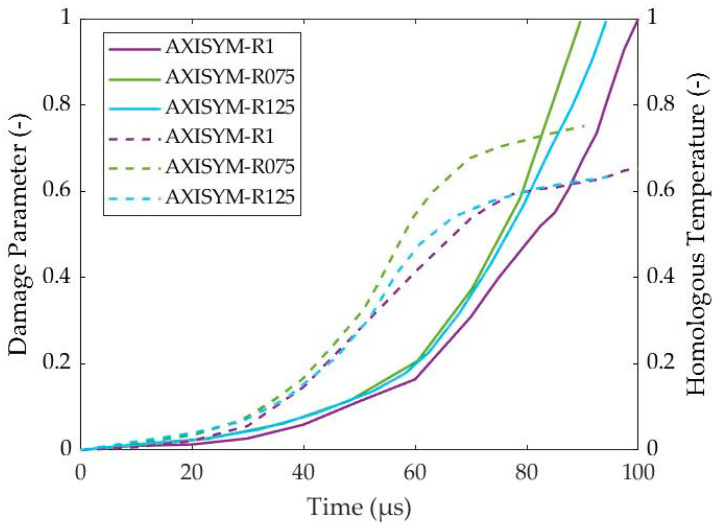
Evolution of damage (solid lines) and homologous temperature (dashed lines) with time for the axisymmetric compression models with different HBR values.

**Figure 22:**
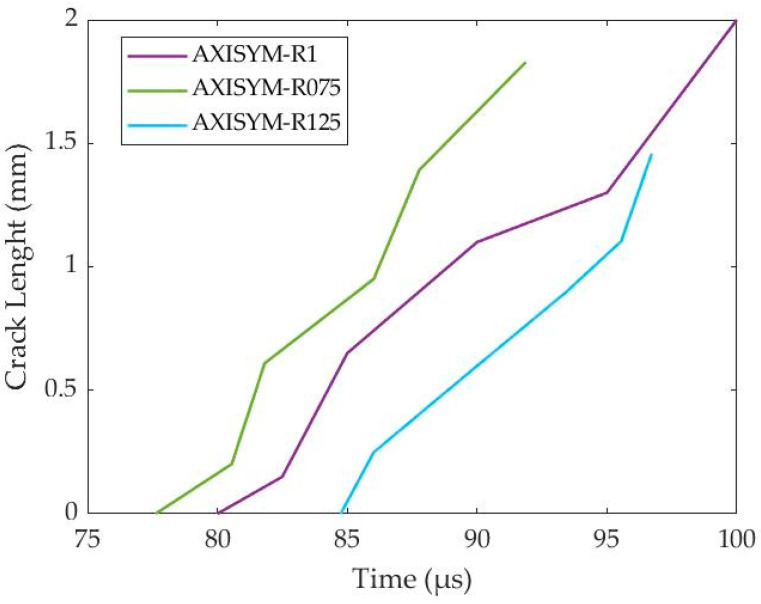
Cracking length with time for the axisymmetric compression models with different HBR values.

Figure 21 demonstrates the competition between damage and homologous temperature evolution and, in consequence, the competition between damage and thermal softening mechanisms. In more detail, the deformation stages before ASB genesis seemed to be dominated by the temperature increase revealing a greater homologous temperature magnitude than the one of damage. In contrast, the later deformation stages after ASB occurrence were shown to be dominated by the superior damage extent. In that way, ASB genesis can be attributed to the thermal softening mechanism, while damage softening can be considered responsible for the later ASB propagation and its transition to fracture at the time regions when the damage parameter was stronger than the homologous temperature. Specifically, the fracture initiated at hot spots inside ASB, which generated small voids due to increased temperature and damage, while cracks were developed next from the voiding coalescence. In fact, four cracks started to develop around the corners of the cross-section and propagate along the softened ASB paths until almost reaching the center of the sample. Finally, Figure 22 depicts the cracking propagation for the axisymmetric models of different HBRs showing that all three simulations reveal almost the same propagating velocity for the cracks, with each crack reaching about 1.5 to 2 mm in final length.

## 5. Conclusions

This work numerically investigated the ASB formation during the dynamic compression of 1045 steel. Plane strain and cylindrical axisymmetric compressions were studied by examining rectangular and cylindrical samples, respectively. Structural-thermal-damage doubly coupled FE models were developed for the numerical simulations in LS-DYNA, implementing MJC thermo-viscoplastic material flow rule and damage criterion together with damage-equivalent stress and strain fields for the full damage coupling. Further, a parametric study on the height-to-base ratio of the sample dimensions was carried out to examine the effect of geometry on the ASB propensity. The main conclusions which were derived from the simulation results can be summarized as follows:

1. Plane strain compression promoted the ASB formation more compared with axisymmetric compression, revealing lower critical strain and earlier ASB onset. The earlier ASB initiation allowed more time for ASB development, resulting in stronger and wider ASBs of higher peak strain and peak temperature inside their core in the case of plane strain compression in contrast to axisymmetric ones where narrower and weaker ASBs formed.
2. In plane strain compression, X-shaped ASBs were initially formed along the diagonals of the cross-section, following the path of maximum shear direction and meeting in the central kink of the sample. However, the central kink was elongated horizontally as deformation progressed, making ASBs receive an S-shape at the final stages. On the contrary, two parabolic ASBs were formed during axisymmetric compression, maintaining their shape even at higher deformation.
3. Early deformation stages of ASB were dominated by the significant temperature rise, while later stages were driven by the more superior damage evolution, revealing that thermal softening precedes damage softening. Therefore, ASB genesis and its early evolution stages were dominated by the more superior thermal softening mechanism, while in contrast damage softening drove the later ASB evolution stages and its transition to fracture. Finally, the fracture often initiates at hot spots via micro-voiding within the ASB core due to the significantly large temperature and the increased damage, and then cracking generation occurs through voiding coalescence.
4. In the case of plane strain compression, the square steel sample of the HBR equal to 1 revealed the greater delay for ASB initiation, resulting in narrower and weaker ASB of lower peak temperature. On the contrary, the PLSTR-R125 simulation revealed the earliest ASB onset, while also higher cracking length and propagating velocity were detected.
5. In the case of axisymmetric compression, the higher the HBR was, the greater the delay on ASB initiation was, revealing higher critical strain and leading to lower peak strain and peak temperature inside ASB. Finally, the HBR did not seem to significantly affect the ASB widening rate and the cracking propagation velocity.
6. The greatest ASB width was obtained at 205 μm during the plane strain compression of the rectangular sample with 1.25 HBR, while in the case of axisymmetric compression, ASB width seemed to decrease with HBR increment. Also, wider heat-affected zones were predicted in all simulations, while ASB width showed a significant increase rate at initial stages from its genesis in contrast to the later stages where widening rate was stabilized.

## Figures and Tables

**Table 1:** Material properties and MJC parameters for AISI 1045 steel.

Material Properties		MJC Constitutive Relation		MJC Damage Rule	
*ρ* (kg/m^3^)	7800	*A* (MPa)	553.1	*D*_1_ (-)	0.06
*Ε* (GPa)	200	*B* (MPa)	600.8	*D*_2_ (-)	3.31
*ν* (-)	0.3	*n* (-)	0.234	*D*_3_ (-)	−1.96
*χ* (-)	0.9	*C* (-)	0.0122	*D*_4_ (-)	0.0018
*C_p_* (J/kgK)	432.6	*m* (-)	1	*D*_5_ (-)	0.58
*α* (μm/m°C)	11	${\dot{\varepsilon }}_{0}$ (1/s)	1	*D_c_* (-)	1
		*T_m_* (K)	1733		
		*T_r_* (K)	293		

**Table 2:** Material properties and MJC parameters for AISI 4340 steel.

Material Properties		MJC Constitutive Relation		MJC Damage Rule	
*ρ* (kg/m^3^)	7830	*A* (MPa)	792	*D*_1_ (-)	0.05
*Ε* (GPa)	205	*B* (MPa)	510	*D*_2_ (-)	3.44
*ν* (-)	0.3	*n* (-)	0.26	*D*_3_ (-)	−2.12
*χ* (-)	0.9	*C* (-)	0.013	*D*_4_ (-)	0.002
*C_p_* (J/kgK)	477	*m* (-)	1.03	*D*_5_ (-)	0.61
*α* (μm/m°C)	11	${\dot{\varepsilon }}_{0}$ (1/s)	1	*D_c_* (-)	1
		*T_m_* (K)	1793		
		*T_r_* (K)	293		

**Table 3:** Comparison between experimental and numerical results for 4340 steel sample.

Description	Experiment [15]	Simulation	Relative Error (%)
Maximum stress *σ_max_* (MPa)	1100	1123	2.09
Yield stress *σ_y_* (MPa)	700	692.7	1.04
Instability point timing *t_cr_* (μs)	318	296	6.92
Critical strain *ε_cr_* (-)	0.518	0.483	6.75
ASB shape	conical/parabolic	conical/parabolic	-

**Table 4:** Developed models for case studies of the simulations.

Model Number	Model Code Name	Strain Field Problem	Height-to-Base Ratio
I	PLSTR-R1	Plane Strain	1
II	PLSTR-R075	Plane Strain	0.75
III	PLSTR-R125	Plane Strain	1.25
IV	AXISYM-R1	Cylindrical Axisymmetric	1
V	AXISYM-R075	Cylindrical Axisymmetric	0.75
VI	AXISYM-R125	Cylindrical Axisymmetric	1.25

**Table 5:** Case studies.

Case Study	Description	Models in Comparison
1	Effect of strain field problem (plane strain vs. cylindrical axisymmetric)	I–IV
2	Effect of height-to-base ratio	I–II–II and IV–V–VI

## Data Availability

The original contributions presented in the study are included in the article, further inquiries can be directed to the corresponding author.
